# Influence of Seeding Ratio, Planting Date, and Termination Date on Rye-Hairy Vetch Cover Crop Mixture Performance under Organic Management

**DOI:** 10.1371/journal.pone.0129597

**Published:** 2015-06-16

**Authors:** Andrew Lawson, Craig Cogger, Andy Bary, Ann-Marie Fortuna

**Affiliations:** 1 Washington State University Puyallup Research and Extension Center, Puyallup, Washington, United States of America; 2 Department of Soil Science, North Dakota State University, Fargo, North Dakota, United States of America; California State University, Fresno, CA, UNITED STATES

## Abstract

Cover crop benefits include nitrogen accumulation and retention, weed suppression, organic matter maintenance, and reduced erosion. Organic farmers need region-specific information on winter cover crop performance to effectively integrate cover crops into their crop rotations. Our research objective was to compare cover crop seeding mixtures, planting dates, and termination dates on performance of rye (*Secale cereale* L.) and hairy vetch (*Vicia villosa* Roth) monocultures and mixtures in the maritime Pacific Northwest USA. The study included four seed mixtures (100% hairy vetch, 25% rye-75% hairy vetch, 50% rye-50% hairy vetch, and 100% rye by seed weight), two planting dates, and two termination dates, using a split-split plot design with four replications over six years. Measurements included winter ground cover; stand composition; cover crop biomass, N concentration, and N uptake; and June soil NO_3_^-^-N. Rye planted in mid-September and terminated in late April averaged 5.1 Mg ha^-1^ biomass, whereas mixtures averaged 4.1 Mg ha^-1^ and hairy vetch 2.3 Mg ha^-1^. Delaying planting by 2.5 weeks reduced average winter ground cover by 65%, biomass by 50%, and cover crop N accumulation by 40%. Similar reductions in biomass and N accumulation occurred for late March termination, compared with late April termination. Mixtures had less annual biomass variability than rye. Mixtures accumulated 103 kg ha^-1^ N and had mean C:N ratio <17:1 when planted in mid-September and terminated in late April. June soil NO_3_^-^-N (0 to 30 cm depth) averaged 62 kg ha^-1^ for rye, 97 kg ha^-1^ for the mixtures, and 119 kg ha^-1^ for hairy vetch. Weeds comprised less of the mixtures biomass (20% weeds by weight at termination) compared with the monocultures (29%). Cover crop mixtures provided a balance between biomass accumulation and N concentration, more consistent biomass over the six-year study, and were more effective at reducing winter weeds compared with monocultures.

## Introduction

Farmers have renewed interest in cover crops that have been shown to improve nitrogen (N) management, enhance crop productivity, suppress weeds, reduce erosion, and improve soil and water quality. The role of cover crops in N supply and weed management is of particular interest to organic growers. Cereal cover crops can recycle residual soil nitrate (NO_3_^-^) that may leach below the rooting zone [1], and legume cover crops can reduce N fertilizer requirements in subsequent crops [2–4]. Cereal cover crops increase soil organic matter via increased C production [5] whereas legumes increase plant available N by atmospheric N fixation [6]. A mixture of legume and cereal cover crops is desirable because mixtures can combine the C and N benefits of cereals and legumes [2,7,8].

In the maritime Pacific Northwest, fall-planted rye (*Secale cereale* L.) or wheat (*Triticum aestivum* L.)-hairy vetch (*Vicia villosa* Roth) cover crop mixtures can produce biomass yields of nearly 5 Mg ha^-1^ by late April, and accumulate up to 100 kg N ha^-1^ in the shoot tissue [9,10]. But, intensive organic vegetable growers in the region are often challenged to fit cover crops into rotations with early and late season vegetable production.

The timing of fall cover crop planting is affected by the harvest of the previous cash crop, the window for reliable establishment, and the number of GDD required for sufficient biomass production. Hairy vetch grows slowly in the fall, often providing minimal winter soil cover [11]. Contrary to hairy vetch, cereal rye can establish quickly when planted by late September in the maritime Northwest, and it can utilize residual soil NO_3_^-^ because rapid root development puts it in contact with soil N [12]. Harvest dates of summer crops vary and unfavorable weather conditions may delay planting beyond the optimal window. Therefore, improved understanding of the effect of delayed planting on stand establishment and biomass yields is needed.

Cover crop N accumulation and biomass composition are affected by spring termination date [9,13–15]. Studies in the eastern USA showed an increase in monoculture hairy vetch biomass of 35 to 61% by delaying the termination date 2 wk from late April to early-mid May, [16,17]. In addition, late termination may be essential when planting in October, to ensure adequate spring biomass and N accumulation [18].

But, delaying the termination date may decrease residue N concentration, increase C:N ratios, and result in higher concentrations of hemicellulose and lignin in residue [15,16], which has an important effect on N release. A delay in rye incorporation from mid March to late April in Denmark decreased net N mineralization and availability to the subsequent crop [14]. For hairy vetch, residue C:N ratio may not be as important if the biomass is terminated before flowering, because a relatively low C:N is maintained in vetch until flowering [19]. The influence of climate and N mineralization rate will affect optimum termination date. Sufficient N accumulation and timely N release are critical aspects in supplying N to the subsequent crop.

Rye-hairy vetch cover crop mixtures generally have higher residue quality than stands of pure rye and have the potential to provide greater plant available N to subsequent crops [8,20,21]. Yet, research addressing the interactive effects of seeding ratio, planting dates, and termination dates is limited.

Winter weed suppression is another primary interest of organic growers. Winter weeds can cause economic losses for summer crops, particularly when facultative (fall and spring germinating) winter annuals such as chickweed (*Stellaria media* L.) survive termination in reduced tillage systems [22]. It has been shown that rye is suitable for winter weed suppression because of rapid establishment and competition for light [23]. Rye was more effective than hairy vetch at suppressing winter weeds in the US Midwest, with rye-hairy vetch mixtures nearly as effective as monoculture rye [22].

Although most cover crop field studies are two to three years in duration, longer-term studies are needed to capture and understand the range and effects of variability among years [24]. This is especially true for cover crop mixtures, where interactions between the members of the mixture can vary, depending on environmental differences from year to year.

In this study, rye-hairy vetch winter cover crops were evaluated over six years for their ability to provide winter ground cover, produce biomass, accumulate N, suppress winter weeds, and increase plant available N in soil. This study was designed to supplement long-term organic vegetable crops systems research in western Washington [25], and is a companion to another study evaluating cover crop N availability and N uptake in organic sweet corn *(Zea mays* L.) [10]. This study specifically explores seeding ratio and planting and termination date effects on rye-hairy vetch cover crops planted after fall harvest. Research questions include: 1) How does planting and termination timing affect cover crop production and characteristics, including ground cover, species composition, biomass, N accumulation, and C:N ratio? 2) How do rye-hairy vetch mixtures compare with monoculture rye and hairy vetch plantings for cover crop production and characteristics across planting and termination dates? 3) How much do cover crop biomass and characteristics vary from year to year, and what are the likely causes of this variability?

## Materials and Methods

### Site description

The experiment was established at the Washington State University Puyallup Research and Extension Center, (47° 11’24” N, 122° 19’48” W; elevation 13 m) in September 2004. The experiment was on land that was placed in organic transition in 2001 and certified organic in accordance with the National Organic Program in 2004. The land was cropped to sudangrass (*Sorghum bicolor* L.) in the summer of 2004 before beginning the experiment. The soil is classified as a Briscot loam (coarse-loamy, mixed, superactive, nonacid, mesic Fluvaquentic Endoaquepts), a deep, somewhat poorly drained soil formed in recent alluvium in the Puyallup Valley of western Washington, USA. The site has a climate typical of the maritime Pacific Northwest, with mild, dry summers and cool, rainy winters. Mean annual precipitation is 1020 mm and mean annual temperature is 11°C. Approximately 75% of precipitation occurs between October and March. Table 1 shows mean monthly temperatures during the cover crop seasons from 2004–2010, collected from an automated weather station 200 m from the field plots.

**Table 1:** Mean monthly temperatures during cover crop season.

	Mean Temperature					
Month	2004–05	2005–06	2006–07	2007–08	2008–09	2009–10
	Degrees C					
**September** ^**a**^	13.0	12.1	14.5	13.4	12.2	14.5
**October**	10.9	11.3	9.6	9.4	9.9	10.3
**November**	6.4	5.0	7.0	6.1	8.9	8.0
**December**	5.2	2.9	4.0	4.6	3.3	1.5
**January**	4.5	5.9	2.6	3.2	4.1	7.4
**February**	3.3	3.7	5.9	5.7	4.1	6.9
**March**	8.4	6.4	8.5	5.8	5.5	7.9
**April**	9.8	9.0	9.6	7.7	9.1	9.6

^a^September means are for 15 September–30 September each year.

### Experimental design

The experiment included four cover crop mixture treatments, two planting dates, and two termination dates, arranged in a randomized complete block split-split plot design, with four replicates. Cover crop mixture treatment was the main plot factor, planting date was the split plot factor, and termination date the split-split plot factor. Main plots measured 9.1 x 6.1 m, with planting date subplots 9.1 x 3.1 m and termination date subplots 9.1 x 1.5 m. Cover crop treatments included cereal rye monoculture (variety not stated), hairy vetch monoculture (variety not stated), and 25:75 and 50:50 (by seed weight) rye-hairy vetch mixtures. Treatments were applied to the same plots each year. The two planting dates were mid-September and early-October and the two termination dates were late-March and late-April. In late March rye was at the tillering stage before stem elongation, and in late April rye was in the stem elongation to early boot stage. Hairy vetch was vegetative at both termination times. In 2005 termination dates were about two weeks later than in 2006–2010.

This experiment was focused on winter cover crops, and no cash crop was grown. Instead, a summer cover crop of sudangrass was planted in June and harvested in August or early September of each year to have a low maintenance crop on the plots during the summer growing season. Because sudangrass yield was initially lower in the monoculture rye plots than in the other treatments (data not shown), starting in 2006 supplemental N was applied to the rye plots each year before planting the sudangrass to avoid increasing N deficiency over time. Nitrogen was applied as feather meal at a rate of 89 kg ha^-1^. No other nutrients were added as baseline soil tests showed pH 6.0 and high levels of P (Bray-1 P: 242 mg kg^-1^ soil) and K (NaHCO_3_ extractable K: 264 mg kg^-1^ soil) [26].

### Management

Cover crops were planted in the fall using a 3.1 m John Deere grain drill, model FB-B, with 15-cm row spacing. All cover crop monocultures and mixtures were seeded at a target rate of 112 kg ha^-1^. Seeding all treatments at the same rate resulted in a seeding rate for hairy vetch that was higher than rates typically used in the Pacific states (28–84 kg ha^-1^) [24, 27]. Hairy vetch seed was inoculated with *Rhizobium leguminosarum* to ensure N fixation. Plots were not irrigated. In the spring after biomass sampling for the late termination date, main plots were flailed (IH-9049 flail mower), and cover crops incorporated using an Imants rotary spader. Sudangrass was planted using a 3.1 m John Deere grain drill at 15-cm row spacing. Supplemental feather meal N was hand broadcast on the monoculture rye plots when sudangrass was planted. Sudangrass was flail mowed and incorporated with the rotary spader in August or early September, and the plots prepared for fall cover crops. Mean biomass of sudangrass incorporated was 2.7 Mg ha^-1^ dry matter. Dates of field activities are listed in Table 2.

**Table 2:** Dates of sampling and field activities.

	Year					
	2004–05	2005–06	2006–07	2007–08	2008–09	2009–10
**Early planting**	15 Sept	20 Sept	14 Sept	18 Sept	17 Sept	14 Sept
**Late planting**	4 Oct	4 Oct	6 Oct	4 Oct	2 Oct	29 Sept
**Ground cover 1**	22 Nov	21 Nov	20 Nov	21 Nov	19 Nov	20 Nov
**Ground cover 2**	25 Jan	30 Jan	23 Jan	8 Feb	2 Feb	25 Jan
**Ground cover 3**	2 Mar	3 Mar	2 Mar	29 Feb	2 Mar	3 Mar
**Early termination**	12 Apr	27 Mar	29 Mar	25 Mar	30 Mar	31 Mar
**Late termination**	6 May	24 Apr	27 Apr	24 Apr	27 Apr	26 Apr
**Residue incorporated**	27 May	26 Apr	30 Apr	1 May	7 May	10 May
**Soil NO_3_-N sampling**	7 June	8 June	25 June	23 June	26 June	24 June
**Sudangrass planting**	14 June	16 June	29 June	16 July	19 June	30 June
**Sudangrass termination**	2 Sept	10 Aug	24 Aug	2 Sept	17 Aug	8 Sept

### Measurements

Winter ground cover was evaluated visually in late November, late January-early February, and early March of each year (Table 2), using three randomly placed 0.25 m^2^ quadrats per plot. Two observers evaluated ground cover in each quadrat as percent of soil covered by cover crop (excluding weeds). The same two observers made the evaluations throughout the study.

In late March and late April, cover crop biomass was measured by harvesting a 6.1 × 0.9 m swath approximately 5 cm above the soil surface of each plot with a small plot forage harvester. The total harvested biomass was weighed, after which a 1 kg subsample was dried at 55°C to calculate biomass yield on a dry weight basis. The remaining harvested biomass was returned to the appropriate plot. Each dried sample was then ground to pass through a 2-mm sieve. Total C and N were measured in the dried and ground samples by dry combustion with a TruSpec CN Carbon/Nitrogen analyzer (Leco, St. Joseph, MI, USA).

Three additional subsamples were randomly collected from representative 0.25 m^2^ quadrats in each plot at each harvest date, and the proportions of rye, hairy vetch, and weeds were determined on a dry weight basis by hand separation and drying at 55°C.

In June of each year, soil samples were collected from the 0 to 30 cm depth for NO_3_^-^-N analysis. Six samples were taken in each plot with a 2.5-cm hand probe, composited, and air-dried at 30°C. Nitrate-N was extracted from 10 g soil samples with 100 mL of 2*M* KCl and determined using the cadmium reduction method [28]. Samples were shaken on a reciprocal shaker for 1 h and then filtered through No. 42 Whatman filters. Aliquots were run on an automated, continuous flow QuikChem 8000 Injection Flow Analysis System (Hach Instruments, Loveland, CO, USA).

### Data analysis

Growing degree days (GDD) from planting through termination were determined using a base temperature of 4°C (Table 3).

**Table 3:** Growing degree days (GDD) between cover crop planting and termination.

	Year						
Planting and termination times	2004–05	2005–06	2006–07	2007–08	2008–09	2009–10	Mean
**Oct-Early**	598	445	519	411	482	663	520
**Oct-Late**	781	571	670	486	618	801	655
**Sept-Early**	765	544	715	533	627	826	668
**Sept-Late**	949	670	867	608	763	964	804
**Mean**	773	558	693	510	623	814	662

GDD use a base temperature of 4°C. Mean daily temperatures calculated as mean of 96 readings collected every 15 minutes from 12:15 AM to midnight.

Cover crop biomass, N uptake, N accumulation, C:N ratio, and stand composition were analyzed with SAS version 9.4, (SAS Institute, Cary, NC, USA) as a split-split plot design using the Mixed Procedure. Ground cover (no termination treatment) and soil NO_3_^-^-N (late termination only) were run as a split-plot because there was no termination split. Cover crop mixtures, planting date, termination date, and year were fixed effects. Biomass and ground cover data were square root transformed and C:N ratio data were inverse transformed to meet assumptions of normality, and means back-transformed for reporting. All other data were analyzed without transformation. Mean separations were done on all significant main effects and interactions. Mean separations were done using LSD with the Tukey-Kramer adjustment at the P = 0.05 significance level. Linear regressions of cover crop biomass vs. GDD were run using the REG procedure in SAS.

## Results and Discussion

Significant treatment main effects and interactions for cover crop, biomass, stand composition, plant N, soil NO_3_^-^-N, and ground cover are summarized in Tables 4 and 5. We present results for cover crop, planting date, and termination date effects and interactions first, followed by year effects and interactions. Discussion of interactions in the text is focused on those with the most biological and practical importance, with the remaining interactions shown in supplemental figures and tables (S1–S12 Figs; S1 and S2 Tables).

**Table 4:** Analysis of variance for cover crop biomass, N, and stand composition as influenced by cover crop, planting date, and termination date treatments and year.

	Biomass	Tissue N concentration	Tissue C:N ratio	Tissue N accumulation	Soil NO_3_^-^-N	Rye stand %	Hairy Vetch stand %	Weeds stand %
**Cover crop mixture**	***	***	***	NS	***	***	***	***
**Planting date**	***	NS	*	***	**	***	NS	***
**Termination date**	***	***	***	***		***	***	***
**Year**	***	***	***	***	***	***	***	***
**Cover crop x Planting date**	***	NS	NS	NS	NS	NS	**	NS
**Cover crop x Termination date**	***	**	***	*		NS	***	NS
**Planting date x Termination date**	***	NS	NS	**		NS	NS	NS
**Cover crop x Year**	***	***	***	***	*	***	***	***
**Planting date x Year**	***	***	***	***	*	***	***	***
**Termination x Year**	***	***	***	***		***	***	***
**Cover crop x Plant** ^**a**^ **x Term**	NS	NS	NS	NS		NS	NS	NS
**Cover Crop x Plant x Year**	NS	*	NS	**	NS	***	NS	NS
**Cover Crop x Term x Year**	*	NS	NS	NS		NS	NS	NS
**Plant x Term x Year**	NS	**	NS	*		NS	NS	NS

*, **, and *** indicate significance at P < 0.05, 0.01, and 0.001, respectively. NS indicates no significant difference at P = 0.05.

^a^Plant and Term refer to planting date and termination date, respectively.

**Table 5:** Analysis of variance for cover crop winter ground cover as influenced by cover crop and planting date treatments and year.

	Cover crop ground cover %		
	November	January	March
**Cover crop mixture**	**	NS	***
**Planting date**	***	***	***
**Year**	***	***	***
**Cover crop x Planting date**	***	*	**
**Cover crop x Year**	***	***	***
**Planting date x Year**	***	***	***
**Cover Crop x Planting date x Year**	*	NS	NS

*, **, and *** indicate significance at P < 0.05, 0.01, and 0.001, respectively. NS indicates no significant difference at P = 0.05.

### Cover crop, planting date, and termination date

#### Biomass

Monoculture rye and rye-hairy vetch mixtures produced greater biomass than monoculture hairy vetch averaged over all planting and termination dates and years (Table 6). Delaying planting from mid-September to early October reduced average biomass by half, and moving termination from late April to late March reduced average biomass by 60%.

**Table 6:** Main effects of cover crop mixture, planting date, and termination date on cover crop biomass, tissue N concentration and accumulation, June soil NO_3_^-^-N, and stand composition at termination 2005–2010.

			Plant tissue N				Stand composition		
Comparison category	Treatment	Biomass	N concentration	C:N ratio	N accumulation	Soil NO_3_^-^-N	Rye	Hairy Vetch	Weeds
		Mg ha^-1^	g N kg^-1^		kg N ha^-1^	mg N kg^-1^ soil	%	%	%
**Cover crop**	100% V^a^	1.4 b	41 a	11 c	61	33 a	0 d	69 a	31 a
	25% R-75% V	2.1 a	32 b	13 b	70	27 ab	43 c	38 b	19 c
	50% R-50% V	2.3 a	30 b	14 b	71	26 b	52 b	27 c	21 bc
	100% R	2.4 a	24 c	18 a	60	18 c	73 a	0 d	27 ab
**Planting date**	September	2.8 a	31	14 a	82 a	28 a	47 a	33	20 b
	October	1.4 b	32	13 b	48 b	23 b	36 b	34	30 a
**Termination date**	Early	1.2 b	35 a	12 b	48 b	-	39 b	31 b	30 a
	Late	3.0 a	28 b	15 a	82 a	26	45 a	35 a	20 b

Means within a comparison category and column followed by different letters are significantly different (P < 0.05) by Tukey-Kramer adjusted LSD.

^a^V and R are hairy vetch and rye in the seeding mix.

**Fig 1:**
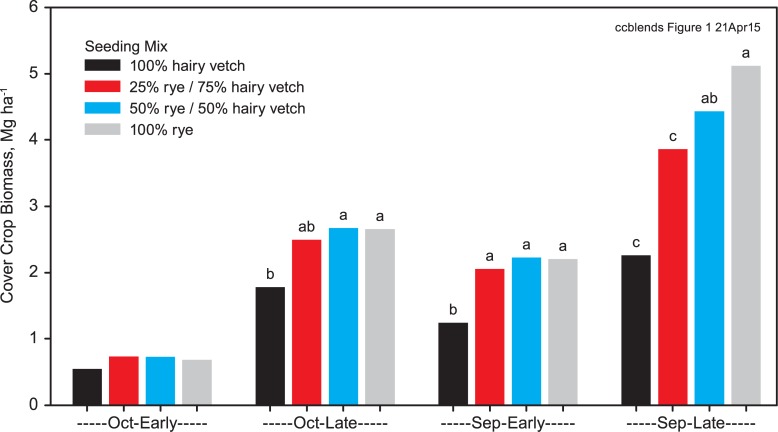
Biomass produced by rye and hairy vetch monocultures and mixtures at October-Early, October-Late, September-Early, and September-Late planting and termination date combinations, averaged over all years. Means within a planting-termination treatment followed by different letters are significantly different (P < 0.05) by Tukey-Kramer adjusted LSD.

Differences in biomass among the cover crop seeding treatments increased with earlier planting and later termination dates as shown by significant cover crop x planting date and cover crop x termination date interactions (Fig 1). There were no significant differences in biomass among cover crop treatments for the October planting and early termination (shortest cover crop growing season). In contrast, rye biomass (5.1 Mg ha^-1^) was more than double hairy vetch biomass (2.3 Mg ha^-1^) for the September planting and late termination (longest cover crop growing season), with the mixtures yielding intermediate biomass (3.9–4.4 Mg ha^-1^). These results indicate a greater rye biomass response to increased growing season under the conditions of our study.

Biomass for the two mixture treatments averaged 4.1 Mg ha^-1^ for the September-April cover crop growing season, which was similar to biomass for cereal-vetch mixtures reported in other studies with similar cool spring climates [9,10,21]. Biomass was lower than typically achieved in milder regions [8,15,24,29], despite later planting and earlier termination in those studies. Planting date x termination date interactions for biomass (and N accumulation) were statistically significant, but not of practical importance (S1 Fig).

#### Winter ground cover

Planting date had the greatest effect on winter ground cover (Table 7). September-planted cover crops had 43% ground cover by late November, compared with only 9% ground cover for October-planted cover crops, averaged over all seeding mixtures and years. Ground cover for the October-planted cover crops remained low throughout the winter, averaging only 27% in early March. The biomass and ground cover data show that delaying fall planting even by two to three weeks can have a profound effect on the functions of cover crops to protect soil and produce biomass.

**Table 7:** Interaction of cover crop mixture and planting date on percent cover crop ground cover assessed in November, January, and March, 2004–05 through 2009–10.

	October planting			September planting		
Cover crop	November	January	March	November	January	March
	% cover crop ground cover			% cover crop ground cover		
**100% V** ^**a**^	10 a	15	29 ab	32 b	35 b	47 b
**25% R-75% V**	9 ab	16	31 a	45 a	49 a	70 a
**50% R-50% V**	8 b	13	26 bc	47 a	46 a	66 a
**100% R**	9 ab	15	23 c	48 a	43 ab	50 b

Means within a column followed by different letters are significantly different (P < 0.05) by Tukey-Kramer adjusted LSD.

^a^V and R are hairy vetch and rye in the seeding mix.

The planting date x cover crop mixture interaction showed that seeding mixture had a smaller effect than planting date on ground cover (Table 7). The September-planted mixtures attained more ground cover than monoculture hairy vetch in November and January, and more ground cover than both monocultures in March. Ground cover was poor for all seeding mixes planted in October.

#### Stand composition

Cover crop seeding mix affected stand composition at termination (Table 6). Weeds comprised less of the mixture biomass (mean 20% weeds by weight) compared with the monocultures (mean 29% weeds by weight), averaged over all planting-termination combinations and years (Table 6). The increased weed suppressive capacity of the mixtures was likely because the mixtures had better vigor than monoculture vetch and more effective architecture for blocking light than monoculture cereals [30]. This was reflected by the greater ground cover of the mixtures apparent by early March (Table 7). Earlier planting and later termination also reduced the proportion of weeds in the biomass (Table 6). September planting and late termination both favored rye over weeds. Late termination also increased the proportion of hairy vetch biomass, but only in the monoculture treatment, as shown by the termination date x cover crop interaction (S1 Table).

Rye outgrew hairy vetch in the cover crop mixtures. The average hairy vetch biomass proportion was equal to one half of the hairy vetch proportion in the seeding mix (27% average hairy vetch biomass for the 50% seeding mix and 38% average hairy vetch biomass for the 75% seeding mix (Table 6)). Others have also observed less vetch biomass than cereal in mixed seedings across a range of environments. Seeding a 30:70 mixture of barley (*Hordeum vulgare* L.) and common vetch (*Vicia sativa* L.) yielded 20–38% vetch in the biomass at termination in central Spain [15], whereas a 40:60 seeding mixture of rye and vetch in coastal California yielded 1–30% vetch in the biomass [29], and a 50:50 rye-hairy vetch seeding mixture in western Washington produced 5–22% vetch in the biomass [10]. The proportion of legumes in the coastal California stands declined between mid-season and termination, indicating strong late season rye growth compared with the legumes [29].

#### Cover Crop Nitrogen

The two rye-hairy vetch mixtures had similar tissue N concentrations averaged over all years and planting-termination combinations, despite a significant difference in the proportion of hairy vetch in the stands (Table 6). Mean tissue N concentration for the mixtures (31 g kg^-1^) was intermediate between monoculture rye (24 g kg^-1^) and monoculture hairy vetch (41 g kg^-1^). C:N ratios showed similar trends (Table 6). Cover crop N accumulation averaged 65 kg ha^-1^, and was not significantly different across seeding mixes, as increasing N concentration with increasing hairy vetch proportion was balanced by decreasing biomass.

Planting date did not influence N concentration, but N accumulation increased by an average of 34 kg ha^-1^ for early compared with late planting, because of greater biomass for the earlier planting (Table 6). No interaction between planting date and cover crop seeding ratio was observed.

**Fig 2:**
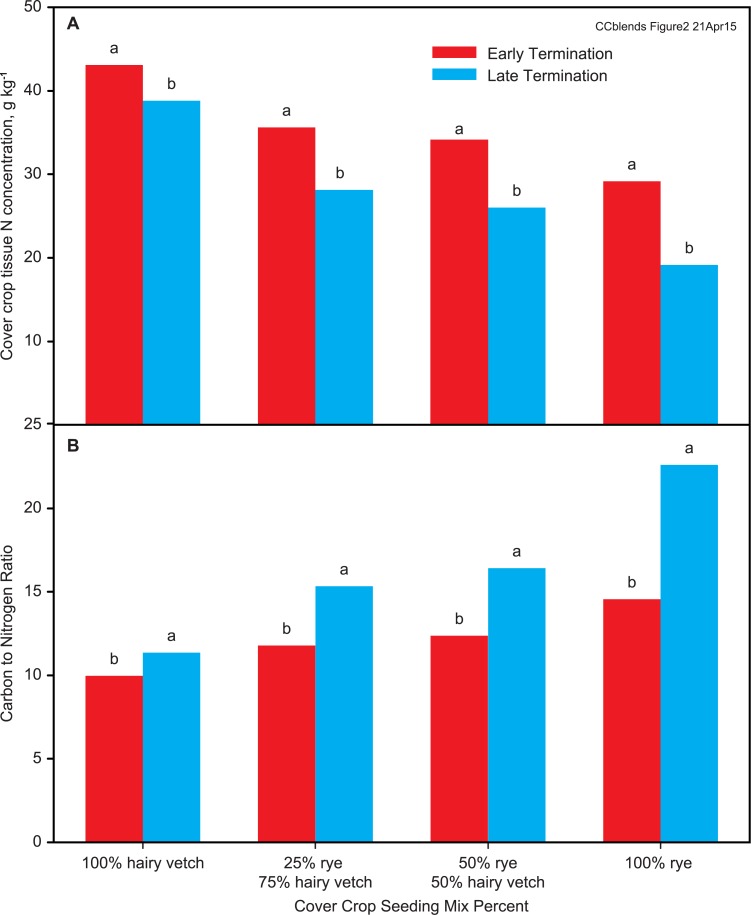
Tissue N concentration (A) and tissue C:N ratio (B) for rye and hairy vetch monocultures and mixtures at early and late termination dates, averaged over all years. Means within a seeding mix treatment followed by different letters are significantly different (P < 0.05) by Tukey-Kramer adjusted LSD.

Late termination reduced plant tissue N concentration for all cover crop treatments, with a significant termination date x cover crop interaction (Fig 2). The average reduction in N concentration between the early and late termination dates was 5 g kg^-1^ for the hairy vetch monoculture, increasing to 8 g kg^-1^ for rye-hairy vetch mixtures, and 10 g kg^-1^ for the rye monoculture (Fig 2a). Despite decreased tissue N concentration, tissue N accumulation increased by 34 kg ha^-1^ compared with early termination, a result of much greater biomass for the late termination date (Table 6).

C:N ratio showed the same trends as N concentration, as expected. Late termination increased C:N ratio for all treatments, with the greatest effect for monoculture rye (cover crop x termination date interaction) (Fig 2b). Rye at the late termination date had a C:N ratio of 23:1 compared with < 17:1 for all other seeding mixture-termination date combinations. The relatively low C:N ratio and high N concentration for monoculture rye in this study compared with other studies [7,8,13,29,31] is because the rye was still vegetative, even at the late termination date. This suggests the potential for greater N availability from monoculture rye and rye-hairy vetch mixtures in this study, compared with studies with more mature rye. A laboratory incubation conducted with cover crop residues of similar maturity, composition, and C:N ratios to this study confirmed net release of N from both monoculture rye and a mixture containing 75% rye and 25% hairy vetch in the biomass [10].

Nitrogen accumulation for the cover crop mixtures for the September planting and late termination dates averaged 103 kg N ha^-1^ (S2 Table). This is greater than reported for cereal-vetch mixtures grown in similar climates for similar lengths of time, where average N accumulation was 50–70 kg ha^-1^ [9,10,21]. Nitrogen accumulation for the September planting and late termination was similar to data from North Carolina (average 94 kg N ha^-1^) [20], but lower than observed in many studies in milder climates, where average N accumulation exceeded 150 kg N ha^-1^ [8,15,24,29].

#### Soil nitrate

Although all cover crop treatments accumulated similar amounts of N, June soil NO_3_^-^-N was greatest after monoculture hairy vetch (33 mg N kg^-1^ soil in the 0 to 30 cm depth), followed by the mixtures (27 mg N kg^-1^ soil) and monoculture rye (18 mg N kg^-1^ soil) (Table 6). Based on a typical bulk density of 1.2 g mL^-1^ [25], mean soil NO_3_^-^-N in the upper 30 cm of the profile ranged from 62 kg ha^-1^ for rye to 97 kg ha^-1^ for the mixtures to 119 kg ha^-1^ for hairy vetch. Soil NO_3_^-^-N was greater than reported by others (35–56 kg N ha^-1^ [21] and 55–65 kg N ha^-1^ [10]) following rye-hairy vetch mixtures in similar soils and climates. September planted cover crops had slightly greater mean June soil NO_3_^-^-N levels than October-planted cover crops (28 vs 23 mg N kg^-1^ soil), with no interaction between cover crop and planting date.

### Year effects and interactions

Cover crop biomass and N accumulation, stand composition, ground cover, and soil NO_3_^-^-N showed the greatest yearly variability averaged over all cover crop, planting, and termination treatments (Table 8). The least variability among years occurred for tissue N concentration and C:N ratio.

**Table 8:** Main effects of year on cover crop biomass, tissue N concentration and accumulation, June soil NO_3_^-^-N, stand composition, and winter ground cover 2004–05 through 2009–10.

		Plant tissue N				Stand composition			Ground cover		
Year	Biomass	N concentration	C:N ratio	N accumulation	Soil NO_3_^-^-N	Rye	Hairy Vetch	Weeds	November	January	March
	Mg ha^-1^	g N kg^-1^		kg N ha^-1^	mg N kg^-1^	%	%	%			
**2004–05**	3.0 a	33 b	14 ab	93 a	37 a	39 d	35 b	27 b	23 c	31 b	46 b
**2005–06**	2.5 b	29 cd	14 b	72 b	33 a	44 c	35 b	21 c	28 b	33 b	46 b
**2006–07**	2.0 d	28 d	-	58 c	26 b	42 cd	29 c	28 b	19 d	18 c	28 c
**2007–08**	1.2 f	35 a	12 c	51 cd	18 c	19 e	45 a	36 a	10 e	12 d	29 c
**2008–09**	1.4 e	34 a	13 c	50 d	36 a	48 b	27 c	26 b	28 b	32 b	43 b
**2009–10**	2.2 c	31 c	15 a	66 b	6 d	59 a	29 c	12 d	34 a	45 a	59 a
**CV (%)**	26	8	9	24	45	31	20	34	34	40	24

Means within a column followed by different letters are significantly different (P < 0.05) by Tukey-Kramer adjusted LSD.

CV is coefficient of variation over years.

#### Biomass, stand composition, and winter ground cover

**Fig 3:**
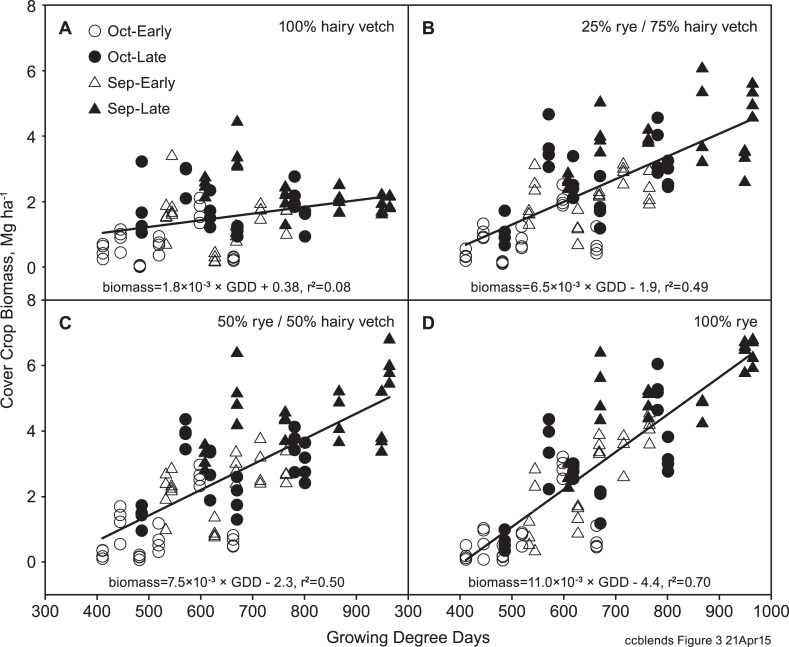
Cover crop biomass vs. cover crop season GDD for rye and hairy vetch monocultures and mixtures. Hairy vetch regression is significant at P < 0.01; all other regressions are significant at P < 0.0001.

Mean annual cover crop biomass production ranged from 1.2 to 3.0 Mg ha^-1^ (Table 8). There was a significant cover crop x year interaction, as annual differences in biomass were affected by GDD and stand composition. The rye monoculture responded most strongly to GDD, with a linear regression slope of 11 kg biomass ha^-1^ GDD^-1^ and r^2^ of 0.70 over the GDD range of this study (Fig 3). The mixtures showed lower response to GDD (6.5–7.5 kg biomass ha^-1^ GDD^-1^; r^2^ = 0.50), and hairy vetch showed the least response (1.8 kg biomass ha^-1^ GDD^-1^; r^2^ = 0.08). The growth response of hairy vetch was less than half that observed in the mid-Atlantic USA, with an average response of 4 kg biomass ha^-1^ GDD^-1^ (r^2^ = 0.56) over a larger GDD range [18].

**Fig 4:**
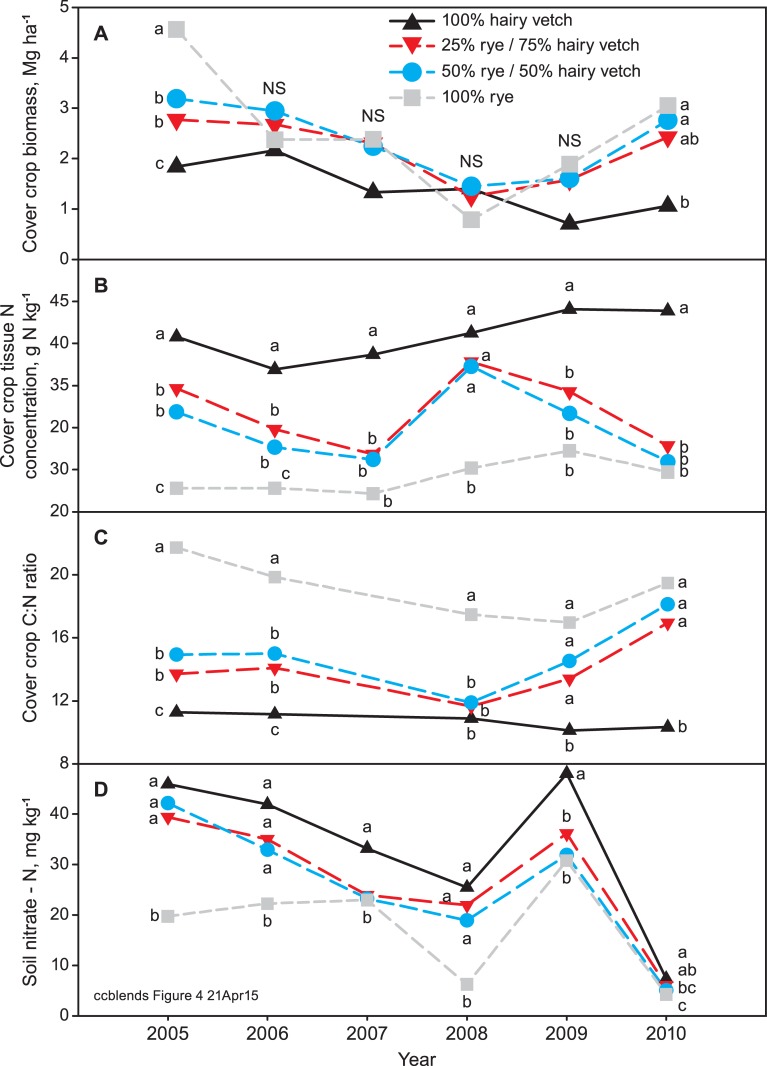
Cover crop x year interactions, showing annual cover crop biomass (A), tissue N concentration (B), C:N ratio (C), and soil NO_3_^-^-N (D) averaged over all planting and termination date treatments. Means within a year followed by different letters are significantly different (P < 0.05) by Tukey-Kramer adjusted LSD.

Stand composition affected biomass in 2007–08 (Table 8, S2 Fig). The rye did not establish or grow well that year, either in the monoculture or mixture plots, resulting in poor ground cover, a stand dominated by hairy vetch and weeds, and low biomass in the rye and mixtures treatments (Fig 4a). This illustrated the importance of a robust rye stand in producing biomass and suppressing weeds. In contrast to 2007–08, the proportion of rye in the stand was unusually high in 2009–10 (Table 8). Despite an overall warm cover crop season on 2009–10, the coldest temperatures of the study occurred in December 2009, with minima of -11 to -12°C on four nights. Winter injury from the cold temperatures likely affected hairy vetch growth relative to rye. Others have observed some winter injury to hairy vetch foliage when absolute winter minimum temperatures reached -11 to -13°C [18].

As a result of degree day and stand composition effects, monoculture rye showed the greatest yearly biomass variability among the cover crop treatments (Fig 4a). The mixtures had similar biomass to the rye averaged over years, but less variability from year to year. The presence of hairy vetch in the mixed stands reduced yearly variability compared with monoculture rye, in part because hairy vetch was less sensitive to degree days than rye under the conditions of this study. Few long term studies are available to compare annual variability in biomass for cereal-legume mixtures and monocultures. Data from a four-year study in western Washington show less biomass variability for a rye-hairy vetch mixture compared with rye and hairy vetch monocultures [21], similar to the results of our study. In contrast, a rye-legume mixture had more variable biomass production than rye monoculture over eight years in central coastal California [24].

Winter ground cover also showed a significant cover crop x year interaction, with the poor rye stand in 2007–08 reducing ground cover in the monoculture rye throughout the winter (S3 Fig).

#### Nitrogen

Cover crop N concentration was less variable over years than biomass, ranging from 28 to 35 g N kg^-1^ dry matter (Table 8) averaged over all cover crops and planting and termination dates. A significant cover crop x year interaction showed that the greatest yearly variability occurred in the mixtures (Fig 4b), and could be explained by differences in stand composition. C:N ratio also had low yearly variability and a significant cover crop x year interaction, with the greatest variability also occurring for the mixtures (Fig 4c). Cover crop N accumulation was more variable over years than cover crop N concentration or C:N, ranging from 50 to 93 kg N ha^-1^ averaged over all seeding mixes, planting, and termination dates (Table 8). Cover crop N accumulation was influenced predominantly by variability in biomass, and showed a similar cover crop x year interaction as biomass, with the greatest year-to-year variability in N accumulation for monoculture rye (S4 Fig).

Soil NO_3_^-^-N showed the largest relative annual range in values, which was the result of a very low mean NO_3_^-^-N level (6 mg N kg^-1^ soil) in 2010 (Table 8). All cover crop treatments (including monoculture hairy vetch) had low soil NO_3_^-^-N in 2010, indicating that low NO_3_^-^ in 2010 was not related to stand composition (cover crop x year interaction) (Fig 4d). The period between cover crop incorporation and soil NO_3_^-^ sampling was unusually wet in 2010, with 164 mm of rainfall, compared with 3 to 81 mm of rainfall during the same period the other years. It is likely that the NO_3_^-^ was lost to leaching below the 30 cm sampling depth, or to denitrification.

The cover crop x year interaction for soil NO_3_^-^–N was also partly the result of fertilizing the rye plots at the time of planting sudangrass beginning in 2006. Soil NO_3_^-^-N levels following rye were lower compared with the mixtures in 2005 and 2006 (Fig 4d), the years before supplemental N was added to the rye plots. Soil NO_3_^-^-N was similar for the rye and mixtures in the following years, except for 2008, which was likely a result of the poor 2007–08 rye stand.

#### Planting and termination date interactions with year

Significant interactions between year and termination date for cover crop biomass, stand composition, and N resulted from variations in the size but not the direction of the termination effects among years (S5 and S6 Figs), and are not of great biological or practical interest. The same is true for most interactions between planting date and year (S7, S8, and S9 Figs), with the exception of hairy vetch composition (S8b Fig). September-planted hairy vetch comprised a greater proportion of the stand at termination than October-planted hairy vetch in 2004–05 and 2007–08, whereas September-planted hairy vetch comprised a smaller proportion of the stand than October-planted hairy vetch in 2006–07.

**Fig 5:**
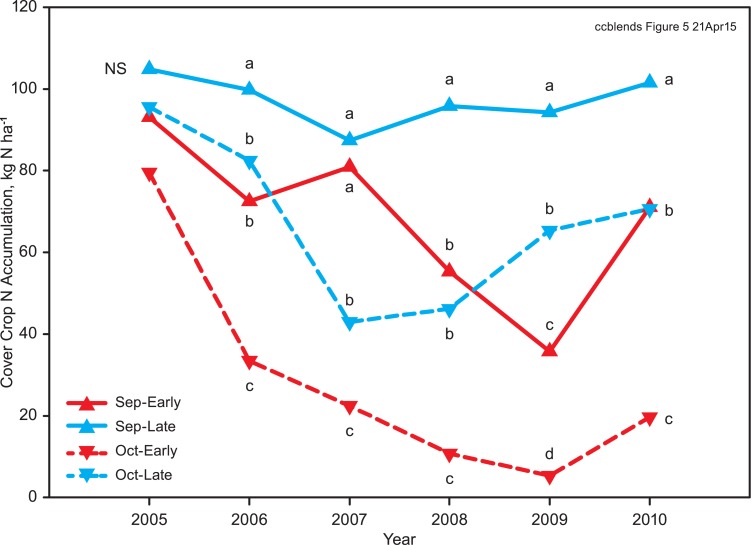
Planting date x termination date x year interaction for cover crop N accumulation averaged over all cover crop mixtures. Means within a year followed by different letters are significantly different (P < 0.05) by Tukey-Kramer adjusted LSD.

A significant three-way interaction for N accumulation among planting date, termination date, and year shows differences in year-to-year variability in N accumulation among the planting and termination date combinations. N accumulation showed less year to year variability for the September planting, late termination (longest cover crop growing season), compared with the other planting-termination combinations (Fig 5).

### Implications for cover crop management in cool maritime climates

#### Cover crop seeding mixture

Rye-hairy vetch seeding mixtures offered several advantages over monocultures of rye or hairy vetch in this study, including better weed suppression than hairy vetch, and similar to better weed suppression than rye. The biomass of the cover crop mixtures was similar to or less than monoculture rye averaged over years, but the mixtures had higher N concentration, lower C:N ratio, and less year-to-year variability in biomass than rye. Despite the dominance of rye in the mixture biomass, the hairy vetch nonetheless added weed suppression and N benefits. The two mixtures performed similarly, indicating no benefit to increasing hairy vetch seeding proportion beyond 50%. The mixtures had more yearly variability in N concentration than either monoculture, a result of variability in stand composition. But, the range in N concentration was not large and would not have a large effect on N management.

The seeding rate of monoculture hairy vetch in this study was higher than typically used by farmers, even in high value cropping systems. Brennan and Boyd [24] found that increasing the seeding rate of cover crops to 3X the normal rate had little effect on cover crop biomass at termination, although it did improve early season biomass production. Although we did not have a seeding rate comparison in this study, mean hairy vetch biomass in the monoculture was lower than for monoculture rye or the mixtures. These results and [24] suggest that the high hairy vetch seeding rate did not skew our results at termination, but also indicate that the high rate was not worth the extra cost. Seeding rates for the mixtures in this study are higher than typically used in the East and Midwest [32], but lower than typical rates for central coastal California [24].

#### Planting and termination dates

Results from this experiment do not support shortening the cover crop season for early or late season cash crop rotations in cool maritime climates in the Pacific Northwest and similar areas. Delaying planting until early October reduced ground cover throughout the winter and reduced spring biomass by an average of 50% and N accumulation by an average of 40% compared with mid-September planting. Although this study did not evaluate planting cover crops before mid-September, others have shown that planting in late August can yield even greater cover crop benefits in the maritime Northwest [9].

Early termination in the spring also substantially reduced cover crop biomass and N accumulation. Although cover crop C:N increased with late termination, cover crops terminated in late April in the maritime Northwest have been shown to have adequate quality for timely N release [10].

Organic farmers do have cover crop options to operate within these timing constraints. Rotations can be planned for fall cover crop planting immediately after harvest of summer crops in late August or September. Relay cover cropping is an alternative for late-harvested crops. [32–35]. Planting relay cover crops between the rows of cash crops gives them an opportunity to become established before the cash crop is harvested. Cover crops are also a good choice where main season crops are planted in May or later, allowing a longer spring period of cover crop growth.

#### Nitrogen contribution

Although this study did not specifically evaluate the N contribution of the cover crops to the following crop, it is useful to consider the N data from this study in the context of other N research. A laboratory incubation estimating N release from a mixture of 75% rye biomass, and 25% hairy vetch biomass at similar growth stage to this study [10], indicated that about 50% of the cover crop N was released over the equivalent of a growing season, or about 50 kg ha^-1^ for the mixtures planted in September and terminated in April in this study. This would affect N management, but would not be enough to meet the N needs of most cash crops.

Monoculture hairy vetch has a higher concentration of N than the mixtures and releases N more rapidly [10], but hairy vetch did not produce enough biomass to provide adequate cash crop N even when planted in mid-September and terminated in late April. Research in Maryland, USA indicated that hairy vetch biomass production of 4 Mg ha^-1^ was needed to supply adequate N for a crop such as corn or tomato (*Solanum lycopersicum* L.) [18]. Hairy vetch biomass in our study averaged only 2.3 Mg ha^-1^ for the September planting and late termination, despite the high seeding rate. Vetches can produce more than 5 Mg ha^-1^ biomass in the maritime Northwest, but termination needs to be delayed until late May to reach that level [31].

Farmers in the maritime Pacific Northwest and similar climates can obtain ground cover, biomass, and N benefits from winter rye and hairy vetch mixtures and monocultures if they allow a sufficiently long cover crop growing season. Nitrogen contributed by the cover crop mixtures in this environment will not be enough to meet the N needs of most cash crops, but can be an important supplemental source of N fertility.

## Supporting Information

S1 Fig Planting date x termination date interaction for cover crop biomass (A) and cover crop N accumulation (B). Means followed by different letters are significantly different (P < 0.05) by Tukey-Kramer adjusted LSD.
(EPS)

S2 Fig Cover crop x year interactions for proportions of rye (A), hairy vetch (B), and weeds (C) in cover crop biomass at termination. Means within a year followed by different letters are significantly different (P < 0.05) by Tukey-Kramer adjusted LSD.
(EPS)

S3 Fig Cover crop x year interactions for ground cover in late November (A), late January (B), and early March (C). Means within a year followed by different letters are significantly different (P < 0.05) by Tukey-Kramer adjusted LSD.
(EPS)

S4 Fig Cover crop x year interaction for cover crop N accumulation. Means within a year followed by different letters are significantly different (P < 0.05) by Tukey-Kramer adjusted LSD.
(EPS)

S5 Fig Termination date x year interactions for cover crop biomass (A), cover crop C:N ratio (B), cover crop tissue N concentration (C), and cover crop N accumulation (D). Means within a year followed by different letters are significantly different (P < 0.05) by Tukey-Kramer adjusted LSD.
(EPS)

S6 Fig Termination date x year interactions for proportions of rye (A), hairy vetch (B), and weeds (C) in cover crop biomass at termination. Means within a year followed by different letters are significantly different (P < 0.05) by Tukey-Kramer adjusted LSD.
(EPS)

S7 Fig Planting date x year interactions for cover crop biomass (A), cover crop tissue N concentration (B), cover crop C:N ratio (C), and cover crop N accumulation (D). Means within a year followed by different letters are significantly different (P < 0.05) by Tukey-Kramer adjusted LSD.
(EPS)

S8 Fig Planting date x year interactions for proportions of rye (A), hairy vetch (B), and weeds (C) in cover crop biomass at termination and June soil NO_3_^-^-N (D). Means within a year followed by different letters are significantly different (P < 0.05) by Tukey-Kramer adjusted LSD.
(EPS)

S9 Fig Planting date x year interactions for ground cover in late November (A), late January (B), and early March (C). Means within a year followed by different letters are significantly different (P < 0.05) by Tukey-Kramer adjusted LSD.
(EPS)

S10 Fig Cover crop x planting date x year interactions for cover crop N tissue concentration (A), cover crop N accumulation (B), proportion of rye in cover crop biomass (C), and ground cover in late November (D). Means within a year followed by different letters are significantly different (P < 0.05) by Tukey-Kramer adjusted LSD.
(EPS)

S11 Fig Plating date x termination date x year interaction for cover crop N concentration. Means within a year followed by different letters are significantly different (P < 0.05) by Tukey-Kramer adjusted LSD.
(EPS)

S12 Fig Cover crop x termination date x year interaction for cover crop biomass. Means within a year followed by different letters are significantly different (P < 0.05) by Tukey-Kramer adjusted LSD.
(EPS)

S1 Table Proportions of hairy vetch in cover crop biomass for different seeding mixtures and planting and termination dates. Means within a column followed by different letters are significantly different (P < 0.05) by Tukey-Kramer adjusted LSD.
(DOCX)

S2 Table Nitrogen accumulation in cover crop treatments at different planting and termination dates. (DOCX)

## References

[1] Brandi-Dohrn FM, Dick RP, Hess M, Kauffman SM, Hemphill DD, Selker JS. Nitrate leaching under a cereal rye cover crop. J Environ Qual. 1997;26: 181–188.

[2] Kuo S, Sainju UM, Jellum EJ. Winter cover cropping influence on nitrogen in soil. Soil Sci Soc Am J. 1997;61: 1392–1399.

[3] Cline GR, Silvernail AF. Effects of cover crops, nitrogen, and tillage on sweet corn. HortTechnology 2002;12: 118–125.

[4] Fortuna A, Blevins RL, Frye WW, Grove J, Cornelius P. Sustaining soil quality with legumes in no-tillage systems. Commun Soil Sci Plant Anal 2008;39: 1680–1699.

[5] Sainju UM, Singh BP, Whitehead WF. Cover crops and nitrogen fertilization effects on soil carbon and nitrogen and tomato yield. Can J Soil Sci. 2000;80: 523–532.

[6] Vaughan JD, Hoyt GD, Wollum AG. Cover crop nitrogen availability to conventional and no-till corn: Soil mineral nitrogen, corn nitrogen status, and corn yield. Commun Soil Sci Plant Anal. 2000;31: 1017–1041.

[7] Ranells NN, Wagger MG. Grass-legume bicultures as winter annual cover crops. Agron J. 1997;89: 659–665.

[8] Sainju UM, Whitehead WF, Singh BP. Biculture legume-cereal cover crops for enhanced biomass yield and carbon and nitrogen. Agron J. 2005;97: 1403–1412.

[9] Odhiambo JJO, Bomke AA. Grass and legume cover crop effects on dry matter and nitrogen accumulation. Agron J. 2001;93: 299–307.

[10] Lawson A, Fortuna A, Cogger CG, Bary AI, Stubbs T. Nitrogen contribution of rye-hairy vetch cover crop blends to organically grown sweet corn. Renew Agr Food Syst. 2012 10.1017/S1742170512000014

[11] Holderbaum JF, Decker AM, Meisinger JJ, Mulford FR, Vough LR. Fall-seeded legume cover crops for no-tillage corn in the humid east. Agron J. 1990;82: 117–124.

[12] Kuo S, Jellum EJ. Long-term winter cover cropping effects on corn (*Zea mays* L.) production and soil nitrogen availability. Biol Fert Soils 2000;31: 470–477.

[13] Clark AJ, Decker AM, Meisinger JJ, McIntosh M. Kill date of vetch, rye, and a vetch-rye mixture. 1. Cover crop and corn nitrogen. Agron J. 1997;89: 427–434.

[14] Thorup-Kristensen K, Dresboll DB. Incorporation time of nitrogen catch crop influences the N effect of the succeeding crop. Soil Use Manage. 2010;26: 27–35.

[15] Alonso-Ayuso M, Gabriel JL, Quemada M. The kill date as a management tool for cover cropping success. PLoS ONE 2014;9(10): e109587 10.1371/journal.pone.0109587 25296333 PMC4190126

[16] Wagger MG. Time of desiccation effects on plant composition and subsequent nitrogen release from several winter annual cover crops. Agron J. 1989;81: 236–241.

[17] Clark AJ, Decker AM, Meisinger JJ, Mulford FR, McIntosh M. Hairy vetch kill date effects on soil-water and corn production. Agron J. 1995;87: 579–585.

[18] Teasdale JR, Devine TE, Mosjidis JA, Bellinder RR, Beste CE. Growth and development of hairy vetch cultivars in the northeastern United States as influenced by planting and harvesting date. Agron J. 2004;96: 1266–1271.

[19] Vaughan JD, Evanylo GK. Corn response to cover crop species, spring desiccation time, and residue management. Agron J. 1998;90: 536–544.

[20] Ranells NN, Wagger MG. Nitrogen release from grass and legume cover crop monocultures and bicultures. Agron J. 1996;88: 777–782.

[21] Kuo S, Jellum EJ. Influence of winter cover crop and residue management on soil nitrogen availability and corn. Agron J. 2002;94: 501–508.

[22] Hayden ZD, Brainard DC, Henshaw B, Ngouajio M. Winter annual weed suppression in rye-vetch cover crop mixtures. Weed Technol. 2012;26: 818–825.

[23] Kruidhof HM, Bastiaans L, Kropff MJ. Ecological weed management by cover cropping: Effects on weed growth in autumn and weed establishment in spring. Weed Res. 2008;48: 492–502.

[24] Brennan EB, Boyd NS. Winter cover crop seeding rate and variety effects during eight years of organic vegetables: I. Cover crop biomass production. Agron J. 2012;104: 684–698.

[25] Pritchett K, Kennedy AC, Cogger CG. Management effects on soil quality in organic vegetable systems in western Washington. Soil Sci Soc Am. J. 2011;75: 605–615.

[26] Horneck DA, Sullivan DM, Owen JS, Hart JM. Soil test interpretation guide. Oregon State University Extension Service Publication EC 1478; 2011.

[27] Sattel R (ed). Using cover crops in Oregon. Oregon State University Extension Service Publication EM 8704; 1998.

[28] Gavlak RG, Horneck DA, Miller RO. Soil, plant, and water reference methods for the Western region 3rd ed Western Regional Ext Publ 125. Windsor, CO Soil Plant Analysis Council; 2005.

[29] Brennan EB, Boyd NS, Smith RF, Foster P. Comparison of rye and legume-rye cover crop mixtures for vegetable production in California. Agron J. 2011;103: 449–463.

[30] Wayman S, Cogger C, Benedict C, Collins D, Burke I, Bary A. Cover crop effects on light, nitrogen, and weeds in organic reduced tillage. Agroecol Sust Food Sys. 2015. (In press).

[31] Sustainable Agriculture Network. Managing cover crops profitably 2nd ed Burlington, VT: Sustainable Agriculture Publications; 2007.

[32] Wayman S, Cogger C, Benedict C, Burke I, Collins D, Bary A. The influence of cover crop variety, termination timing, and termination method on mulch, weed cover, and soil nitrate in reduce-tillage organic systems. Renew Agr Food Syst. 2014 10.1017/S1742170514000246

[33] Hively WD, Cox WJ. Interseeding cover crops into soybean and subsequent corn yields. Agron J. 2001;93: 308–313.

[34] Baributsa DN, Foster EF, Thelen KD, Kravchenko AN, Mutch DR, Ngouajio M. Corn and cover crop response to corn density in an interseeding system. Agron J. 2008;100: 981–987.

[35] Bich AD, Reese CL, Kennedy AC, Clay DE, Clay SA. Corn yield in not reduced by mid-season establishment of cover crops in Northern Great Plains environments. Crop Manage. 2014 10.2134/CM-2014-0009-RS

