# Supplementary material for: Influence of Seeding Ratio, Planting Date, and Termination Date on Rye-Hairy Vetch Cover Crop Mixture Performance under Organic Management
Source: PLoS One. 2015 Jun 16;10(6):e0129597. doi: 10.1371/journal.pone.0129597 (PMC4469325; doi:10.1371/journal.pone.0129597)
Supplement: S1 Table — Means within a column followed by different letters are significantly different (P < 0.05) by Tukey-Kramer adjusted LSD. (DOCX) [file pone.0129597.s013.docx]

**S1 Table. Proportions of hairy vetch in cover crop biomass for different seeding blends and planting and termination dates**.

|  | **Percentage of hairy vetch in seeding blend** | | |
| --- | --- | --- | --- |
| **Planting and Termination Timing** | **50** | **75** | **100** |
|  | Percentage of Hairy Vetch in Biomass | | |
| October-Early | 26 | 42 | 59 c |
| October-Late | 29 | 42 | 71 ab |
| September-Early | 25 | 35 | 65 bc |
| September-Late | 27 | 33 | 79 a |
|  | p=0.31 | p=0.06 | p=0.002 |

Means within a column followed by different letters are significantly different (P < 0.05) by Tukey-Kramer adjusted LSD.
