# Supplementary material for: Influence of Seeding Ratio, Planting Date, and Termination Date on Rye-Hairy Vetch Cover Crop Mixture Performance under Organic Management
Source: PLoS One. 2015 Jun 16;10(6):e0129597. doi: 10.1371/journal.pone.0129597 (PMC4469325; doi:10.1371/journal.pone.0129597)
Supplement: S2 Table — (DOCX) [file pone.0129597.s014.docx]

**S2 Table. Nitrogen accumulation in cover crop treatments at different planting and termination dates.**

| **Seeding Mix** | **October-Early** | **October-Late** | **September-Early** | **September-Late** |
| --- | --- | --- | --- | --- |
|  | N accumulation kg ha^-1^ | | | |
| **100% vetch** | 27 | 69 | 56 | 89 |
| **25% vetch-75% rye** | 30 | 73 | 75 | 100 |
| **50% vetch-50% rye** | 31 | 71 | 73 | 107 |
| **100% rye** | 25 | 56 | 68 | 93 |
|  | p=0.39 | p=0.07 | p=0.12 | p=0.07 |
